# Text-Mining in Long-Term Care: Exploring the Usefulness of Computer-Aided Analyzing Methods

**DOI:** 10.1093/geroni/igab046.1993

**Published:** 2021-12-17

**Authors:** Sil Aarts, Coen Hacking, Hilde Verbeek, Jan Hamers, Katya Sion

**Affiliations:** 1 Maastricht university, Maastricht, Limburg, Netherlands; 2 Maastricht University, Maastricht, Limburg, Netherlands

## Abstract

In nursing homes, narrative data are collected to evaluate quality of care as perceived by residents or their family members. This results in a large amount of textual data which exceeds the capability of humans to analyse it. This study aims to explore the usefulness of text-mining approaches regarding narrative data gathered in a nursing home setting. Data has been collected as part of the project ‘Connecting Conversations’: assessing experienced quality of care by conducting individual interviews (n=125) with residents of nursing homes, family members and care professionals. Several pre-processing steps were applied to the textual data. Finally, a variety of text-mining analyses were conducted: individual and bigram word frequencies, correlation analysis and sentiment analysis. A survey was conducted to establish a sentiment analysis model tailored to text collected in long-term care for older adults. Residents, family members and care professionals uttered respectively 285, 362 and 549 words per interview. Word frequency analysis showed that words that occurred most frequently in the interviews are often positive. Although there are some differences in wording such as the use of ‘mother’ and ‘breakfast’, correlation analysis displayed that similar words are used by all three groups to describe quality of care. The majority of interviews displayed a neutral sentiment. Care professionals are more diverse in their sentiment than residents and family members: while some express a more positive sentiment, others express more negativity. This study demonstrates the usefulness of text-mining to extend our knowledge regarding quality of care in a nursing home setting.

